# Validity of type 2 diabetes diagnosis in a population-based electronic health record database

**DOI:** 10.1186/s12911-017-0439-z

**Published:** 2017-04-08

**Authors:** Conchi Moreno-Iribas, Carmen Sayon-Orea, Josu Delfrade, Eva Ardanaz, Javier Gorricho, Rosana Burgui, Marian Nuin, Marcela Guevara

**Affiliations:** 1 Navarra Public Health Institute, Leyre 15, 31003 Pamplona, Spain; 2 Research Network for Health Services in Chronic Diseases (REDISSEC), Madrid, Spain; 3 Navarra Institute for Health Research (IdiSNA), Pamplona, Spain; 4 Department of Preventive Medicine and Public Health, Navarra Hospital Complex, Pamplona, Spain; 5 Department of Preventive Medicine and Public Health, University of Navarra, Pamplona, Spain; 6 Biomedical Research Center Network for Epidemiology and Public Health (CIBERESP), Madrid, Spain; 7 Department of Health, Navarra Regional Government, Pamplona, Spain; 8 Primary Healthcare Directorate, Navarra Health Service, Pamplona, Spain

**Keywords:** Validity, Type 2 diabetes mellitus, Incidence, Electronic health records, Primary care

## Abstract

**Background:**

The increasing burden of type 2 diabetes mellitus makes the continuous surveillance of its prevalence and incidence advisable. Electronic health records (EHRs) have great potential for research and surveillance purposes; however the quality of their data must first be evaluated for fitness for use. The aim of this study was to assess the validity of type 2 diabetes diagnosis in a primary care EHR database covering more than half a million inhabitants, 97% of the population in Navarra, Spain.

**Methods:**

In the Navarra EPIC-InterAct study, the validity of the T90 code from the *International Classification of Primary Care, Second Edition* was studied in a primary care EHR database to identify incident cases of type 2 diabetes, using a multi-source approach as the gold standard. The sensitivity, specificity, positive predictive value, negative predictive value and the kappa index were calculated. Additionally, type 2 diabetes prevalence from the EHR database was compared with estimations from a health survey.

**Results:**

The sensitivity, specificity, positive predictive value and negative predictive value of incident type 2 diabetes recorded in the EHRs were 98.2, 99.3, 92.2 and 99.8%, respectively, and the kappa index was 0.946. Overall prevalence of type 2 diabetes diagnosed in the EHRs among adults (35–84 years of age) was 7.2% (95% confidence interval [CI] 7.2–7.3) in men and 5.9% (95% CI 5.8–5.9) in women, which was similar to the prevalence estimated from the health survey: 8.5% (95% CI 7.1–9.8) and 5.5% (95% CI 4.4–6.6) in men and women, respectively.

**Conclusions:**

The high sensitivity and specificity of type 2 diabetes diagnosis found in the primary care EHRs make this database a good source for population-based surveillance of incident and prevalent type 2 diabetes, as well as for monitoring quality of care and health outcomes in diabetic patients.

## Background

According to the World Health Organization (WHO), type 2 diabetes mellitus is caused by the body’s ineffective use of insulin and is mainly the result of excess body weight and physical inactivity. In 2014 it was estimated that the prevalence of type 2 diabetes around the world was 9% among adults over 18 years old, and in 2012 diabetes was responsible for1.5 million deaths [1]. Observational studies have found that diabetes is associated with an increased risk of cardiovascular diseases and all-cause mortality [2–4].

The WHO recommends continuous surveillance of prevalence and incidence of the most common noncommunicable diseases: cardiovascular diseases, cancer, respiratory diseases and diabetes [5]. Several approaches have been used to monitor type 2 diabetes, including the use of mortality statistics, surveys with or without laboratory tests, diabetes registers, electronic health records (EHRs), diabetes medications prescriptions, claims, diabetes diagnosis in hospital discharge and laboratory tests. In recent years, some algorithms have been developed for ascertaining type 2 diabetes in adults and children using administrative and clinical databases, for example in Canada [6–10], the UK [11] and Spain [12–14], among others. The use of EHR data for surveillance does not require bespoke data collection or patient recruitment [15], provides data for large populations and could be cost-effective [16]. However, it is necessary to evaluate the validity of this data, obtained for clinical reasons, before using them for surveillance or research.

In Navarra, a region with 640,000 inhabitants, a single primary care EHR database covers >97% of the population. This database is administered by a software based in OMI-AP [17] and is structured around a list of episodes (problems in the bio-psycho-social sphere, reasons for consultation, etc.) coded according to the *International Classification of Primary Care, Second Edition* (*ICPC-2*) [18]. Coding gathered in this database distinguishes type 2 diabetes (T90) from type 1 diabetes (T89), impaired fasting glycemia (A91 descriptive term “impaired fasting glycemia”), glucose intolerance (T99 descriptive term “Glucose intolerance”) and gestational diabetes (W85).

The aim of this study was to assess the validity of the primary care EHR data for the surveillance of incidence and prevalence of type 2 diabetes in the Navarra population.

## Methods

The ability of EHR to accurately identify incident cases of diagnosed type 2 diabetes was investigated in a cohort from Navarra that had been included in a large prospective type 2 diabetes case-cohort study nested within the European Prospective Investigation into Cancer and Nutrition (EPIC-InterAct study) [19]. This is a large multi-center study to investigate how genetic and lifestyle behavioral factors, particularly diet and physical activity, interact in their influence on the risk of developing type 2 diabetes. The Navarra EPIC cohort included 8084 participants (3908 men and 4176 women) aged 45–65 years at the time of enrollment (1992–1995). Most of the participants were blood donors (75%), and the rest were civil servants and general population. More detailed information about the EPIC study methods have been described elsewhere [20, 21]. A sensitive approach was used with the aim of identifying all potential incident diabetes cases between the recruitment and December 2007 using multiple sources: self-reported diabetes or use of diabetes medication in a follow-up survey carried out 3 years after recruitment, diabetes diagnosis in the hospital discharge databases, type 2 diabetes (T90), type 1 diabetes (T89) and T99 (descriptive term “glucose intolerance”) diagnosis in primary care EHRs, prescription of antidiabetic drugs and cause-of-death register. A team of trained health professionals reviewed the clinical data to verify if the cases fulfilled the criteria proposed by the American Diabetes Association (ADA) in 2003: 1) Symptoms of diabetes (e.g.: polyuria, polydipsia, and unexplained weight loss) plus casual plasma glucose concentration ≥200 mg/dL (11.1 mmol/L). Or 2) Fasting plasma glucose (FPG) ≥126 mg/dL (7.0 mmol/L). Or 3) 2-h PG ≥200 mg/dL (11.1 mmol/L) during an oral glucose tolerance test (OGTT). In the absence of unequivocal hyperglycemia with acute metabolic decompensation, these criteria should be confirmed by repeat testing on a different day [22]. We excluded from the analysis 262 prevalent type 2 diabetes cases at recruitment, 5 participants who died before 2003 when EHR use was universalized in Navarra and 130 without primary care EHRs, resulting in a final sample of 7687 (3654 men and 4033 women).

The completeness of prevalence type 2 diabetes data recorded in EHRs was studied using “comparison of rates” [11, 23] methodology. To this end, type 2 diabetes prevalence in primary care EHR database in 2005 was compared with the estimations obtained from a health survey carried out in a census sample from the Navarra adult population in 2003 [24]. With the exception of <3% of the population (which was covered by private health insurance), the EHR database encompassed the entire Navarra population’s use of primary care centers.

Briefly, the aim of this survey was to estimate the prevalence of several cardiovascular risk factors including diabetes. After a response rate of 71%, self-reported diabetes prevalence was estimated in 4354 participants between 35 and 84 years of age. Data from surviving registered patients diagnosed with type 2 diabetes (ICPC-2, code T90) in June 2005 (*n* = 22,313) served to estimate type 2 diabetes prevalence in the EHR database that was compared with the health survey estimations to assess completeness and determine the level of under-reporting or over-reporting.

### Statistical analyses

After the multi-source search and verification against medical records, EPIC cohort participants were categorized as having or not having an incident type 2 diabetes diagnosis. We compared T90-coded data in the EHRs against this gold standard to calculate sensitivity, specificity, positive predictive value (PPV) and negative predictive value (NPV) with their 95% confidence intervals (CI). The kappa index was also calculated. This validation method has been used successfully by other authors [12, 14, 25, 26]. The sensitivity was defined as the proportion of cases with T90 codes in the EHR database among those who were true incident type 2 diabetes cases according to the gold standard. The specificity was defined as the proportion of cases without T90 codes in the EHRs among those who were not type 2 diabetes cases according to the gold standard. The PPV was defined as the probability that a patient with a T90 code in the EHRs would be considered a true type 2 diabetes case according to the gold standard, and the NPV was defined as the probability that a patient without type 2 diabetes code would not meet the criteria to be considered a type 2 diabetes case according to the gold standard.

Additionally, we estimated the accuracy of the date of diagnosis registered in the EHRs via comparison with the real date of diagnosis in those patients who had been diagnosed between 2003 and 2006.

To evaluate the completeness of the type 2 diabetes diagnosis reported in the EHR database, the population was divided into five age groups as follows: 35–44 years, 45–54 years, 55–64 years, 65–74 years and 75–84 years. The prevalence of type 2 diabetes and its 95% CI in each sex-age group was compared with the prevalence of self-reported type 2 diabetes in the health survey. Age-adjusted prevalence in men and women 35–84 years of age was estimated using the age distribution of the Navarra population as a reference.

All statistical analyses were performed with the statistical software STATA/SE (version 12.0).

## Results

A total of 1285 charts of potential diabetes cases were reviewed and 598 cases of type 2 diabetes were confirmed in the EPIC-InterAct cohort. The T90 code for type 2 diabetes in the EHR database had a high sensitivity (98.2%), specificity (99.3%), PPV (92.2%) and NPV (99.8%) (Tables 1 and 2). The degree of global agreement measured with the kappa index was very high: ƙ = 0.946 (*p* <0.001).

**Table 1:** Measures of validity of type 2 diabetes code (ICPC-2, T90) in a primary care EHR database. Navarra EPIC-InterAct cohort

	Gold standard (diagnosis from multiple sources)		Total
Information of EHR	T2DM yes	T2DM no	
Code T90 yes	(A) TP True cases correctly identified in EHR	(B) FP Non-cases wrongly coded in EHR	(A + B)
	587	50	637
Code T90 no	(C) FN True cases not identified in EHR	(D) TN True non-cases correctly identified in EHR	(C + D)
	11	7039	7050
Total	(A + C)	(B + D)	(A + B + C + D)
	598	7089	7687

Sensitivity: A/(A + C); specificity: D/(B + D); positive predictive value: A/(A + B); negative predictive value: D/(C + D)

**Table 2:** Validity of type 2 diabetes code (ICPC-2, T90) in a primary care EHR database. Navarra EPIC-InterAct cohort

Sensitivity % (95% CI)	Specificity % (95% CI)	PPV % (95% CI)	NPV % (95% CI)	Kappa index (95% CI)
98.2 (96.7–99.1)	99.3 (99.1–99.5)	92.2 (89.8–94.1)	99.8 (99.7–99.9)	0.946 (0.933–0.960)

*Abbreviations*: *ICPC-2, International Classification of Primary Care*, Second Edition, *PPV* positive predictive value, *NPV* negative predictive value

The difference between the date of diagnosis reported in the EHRs (date of the T90 code) and the date of diagnosis according to the gold standard was less than 12 months in 3 out of 4 patients (Table 3).

**Table 3:** Time lag of T90 code date in comparison with diagnosis date. Navarra EPIC-InterAct cohort (cases of type 2 diabetes diagnosed between 2003 and 2006)

Time lag (months)	*N*	%
<12	154	75.5
12–23	31	15.2
24–35	13	6.4
≥36	6	2.9
Total	204	100.0

**Fig. 1:**
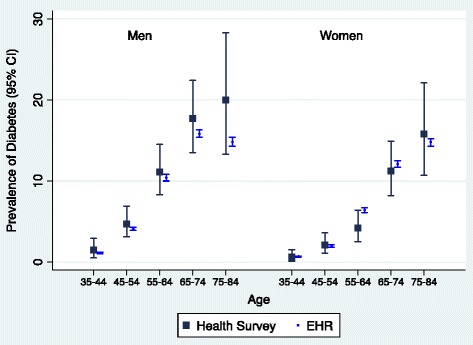
Comparison of type 2 diabetes prevalence estimated by the health survey and prevalence registered in primary EHR database by sex and age

Type 2 diabetes prevalence estimates based on EHR data were comparable to those obtained from the health survey in all age groups. Overall prevalence of type 2 diabetes diagnosed in the EHRs among adults (35–84 years) was 7.2% (95% CI 7.2–7.3) and 5.9% (95% CI 5.8–5.9) in men and women, respectively, very similar to the prevalence estimated from the health survey: 8.5% (95% CI 7.1–9.8) in men and 5.5% (95% CI 4.4–6.6) in women (Table 4 and Fig. 1).

**Table 4:** Comparison between type 2 diabetes prevalence registered in the primary care EHR database and self-reported prevalence from a health survey

	Diabetes prevalence in 2003 (health survey)	Population in primary care EHR database in 2005	Expected cases in 2005 according to the health survey prevalence	Registered cases in primary care EHR (code T90) in June 2005		Ratio between registered and expected cases
	%	*n*	*n*	*n*	%	%
Men						
35–44 years	1.5	48,244	723	525	1.1	72.6
45–54 years	4.7	38,169	1803	1568	4.1	87.0
55–64 years	11.1	30,974	3442	3226	10.4	93.7
65–74 years	17.7	23,003	4064	3643	15.8	89.7
75–84 years	20.3	15,567	3166	2311	14.8	73.0
35–84 years		155,957	13,198	11,273		85.4
Age-adjusted prevalence (95% CI%)	8.5				7.2	
	(7.1–9.8)				(7.2–7.3)	
Women						
35–44 years	0.6	44,431	269	290	0.7	107.7
45–54 years	2.1	36,546	771	708	1.9	91.8
55–64 years	4.2	30,832	1282	1970	6.4	153.7
65–74 years	11.2	25,855	2904	3121	12.1	107.5
75–84 years	16	22,261	3557	3288	14.8	92.5
35–84 years		159,925	8783	9377		106.8
Age-adjusted prevalence (95% CI)	5.5				5.9	
	(4.4–6.6)				(5.8–5.9)	

*Abbreviations*: *EHR* electronic health record

## Discussion

Most of the type 2 diabetes diagnoses in the primary care EHRs were well recorded when compared against the “gold standard,” showing a high sensitivity, specificity, PPV and NPV, and also a very high agreement. Moreover, this study showed that the information obtained from the EHRs provides a good estimation of type 2 diabetes prevalence in population with 35–84 years of age.

The incidence study in the EPIC-InterAct cohort showed that code T90 has a high sensitivity (98.2%), higher than that published in the outpatient records from a large health system, 79% [25]. The percentage of false negative cases in our study was 1.8%, and they corresponded mainly to cases that were coded as type 1 diabetes, impaired fasting glycemia or glucose intolerance. Similar sensitivity, 99.5%, was found in one study published in Spain using an internal validation method [14]. Another study conducted in Spain by Gil-Montalban et al. [12] using a similar approach as ours found a sensitivity of 83.5%. The sensitivity of administrative data ranged from 46 to 97% (median 81.5%) in a systematic review conducted by Saydah et al. [26].

In our study, we found a high specificity for the diagnosis of type 2 diabetes: 99.3%. The percentage of false positive cases was only 0.7% and most of them corresponded to patients that had impaired fasting glycemia, patients with adverse effects to some medications that cause hyperglycemia and type 1 diabetes cases. Other studies conducted in Spain have also reported specificities over 98% for the primary care EHRs [12, 14]. The review of studies based on administrative data carried out by Saydah et al. [26] found that the specificity was consistently high and ranged from 95 to 100% (median 99%).

The PPV and NPV that we found in our study were 92.2 and 99.8%, respectively. These were higher than the values observed by Gil-Montalban et al. [12] at 78.5 and 98.7%, and very similar to those found by Burgos-Lunar et al. [14] at 91.2 and 99.9% for PPV and NPV, respectively. Additionally the review of Saydah et al. [26] found that the PPV ranged from 60 to 98% (median 92%). Finally, the agreement found between the T90 codes in the EHRs and the gold standard was high at ƙ = 0.94 (*p* >0.001), as in other studies conducted in Spain, 0.99 and 0.79 (14,12). Additionally, Saydah et al. [26] found that the kappa index of the studies included in their review ranged from 67 to 96% (median 83%).

The prevalence of type 2 diabetes that we found in Navarra (7.2 and 5.9% in men and women ≥35 years, respectively) were quite similar to those found in adults over 30 years of age from Madrid, Spain (8.5 and 5.9% in men and women, respectively) registered in the primary care EHR database [12]. Additionally, the standardized prevalence of diabetes estimated with data from Spain’s 2006/2007 National Health Survey (NHS) [27] was 6.6% (CI95% 6.1–7.2) among men and 5.6% (CI95% 5.2–6.0) among women. The prevalence of the NHS was lower that the ones we found in our EHRs because they included adults above 16 years of age and our prevalence was calculated with older people (≥35 years), therefore our results are not fully comparable.

One limitation of the present study is the fact that the survey conducted in 2003 did not register the specific type of diabetes (1 or 2). However, the prevalence of type 1 diabetes represents only around 4% of total diabetes cases in Spain [28]. The strengths of the study are the exhaustive examination of clinical data in each case of potential type 2 diabetes identified in multiples source, the use of the whole population of Navarra for the prevalence study validation and the large number of participants included for the incidence validation study.

To the best of our knowledge, this is the first study that validates diabetes diagnosis in primary care EHRs using a multi-source approach including self-reported diabetes, hospital discharge diagnoses, drug prescription records, cause-of-death registers and several diabetes-related codes from the EHRs. Moreover, all potential diabetes cases were reviewed to confirm their fulfillment of the criteria proposed by the ADA in 2003. Implementation of algorithms that include other variables from the EHRs, especially prescription of antidiabetic drugs, would identify cases that need review to improve the accuracy of estimation as has been shown in a recent study from Spain [28].

The validation methodology used in this study might also be useful for validating EHR diagnosis of those diseases that need continuous surveillance of prevalence and incidence such as: cardiovascular diseases, cancer and respiratory diseases.

## Conclusions

With the results of this validation study, we can conclude that the accuracy and completeness of type 2 diabetes diagnoses in the primary care EHR database proved it to be a valid source for epidemiological surveillance and quality care monitoring in our population.

## Abbreviations

ADA: American Diabetes Association
CI: Confidence intervals
EHR: Electronic health record
EPIC: European Prospective Investigation into Cancer and Nutrition
ICPC-2: *International Classification of Primary Care, Second Edition*
NPV: Negative predictive value
PPV: Positive predictive value
WHO: World Health Organization
